# Gene-specific FACS sorting method for target selection in high-throughput amplicon sequencing

**DOI:** 10.1186/1471-2164-11-140

**Published:** 2010-02-26

**Authors:** Julia Sandberg, Marten Neiman, Afshin Ahmadian, Joakim Lundeberg

**Affiliations:** 1 School of Biotechnology, Division of Gene Technology, AlbaNova University Center, Royal Institute of Technology, 106 91 Stockholm, Sweden

## Abstract

**Background:**

In addition to shotgun sequencing, next generation sequencing has been shown to be suitable for deep sequencing of many specific PCR-amplified target genes in parallel. However, unspecific product formation is a common problem in amplicon sequencing since these fragments are difficult to fully remove by gel purification, and their presence inevitably reduces the number of mappable sequence reads that can be obtained in each sequencing run.

**Results:**

We have used a novel flow cytometric sorting approach to specifically enrich Roche/454 DNA Capture beads carrying target DNA sequences on their surface, and reject beads carrying unspecific sequences. This procedure gives a nearly three-fold increase in the fraction of informative sequences obtained. Presented results also show that there are no significant differences in the distribution or presence of different genotypes between a FACS-enriched sample and a standard-enriched control sample.

**Conclusions:**

Target-specific FACS enrichment prior to Roche/454 sequencing provides a quick, inexpensive way of increasing the amount of high quality data obtained in a single sequencing run, without introducing any sequence bias.

## Background

Next-generation sequencing approaches have proved to be highly valuable for the analysis of genomes [1-3], transcriptomes [4-6] and transcription factor binding sites [7]. In addition, they are useful for analysis of specific target regions, notably in ultra-deep sequencing of amplicons from tumor biopsies for studying the genetic changes or clonal expansion of cancer [8,9]. Another widely used application of amplicons is in the development of generic targets for many bacterial species in 16S rRNA metagenomics projects [10,11]. A commonly used approach in amplicon sequencing is to introduce sequencing handles by extending the amplicon with a protruding sequence, and thus facilitate next generation sequencing with the standardized sets of primer sequences recommended by the manufacturers of the sequencing equipment. A sample identification tag is also often introduced at the same time.

However, we have observed that the introduction of Genome Sequencer primer handle sequences (denoted A, B) can result in the formation of short unspecific products; fragments that amplify more readily than target sequences and thus threaten to severely reduce the frequencies of target sequence reads in a sample. Unspecific products can be removed, to some extent, by gel purification if there is sufficient size difference between them and the target sequences, or alternatively the handle sequences can be omitted in the PCR and the A, B handle sequences can be added through more laborious linker ligation.

We have previously demonstrated that Fluorescence Activated Cell Sorting (FACS) can be used for the enrichment of DNA-covered beads in sample preparation for Roche/454 sequencing. This is a quick, inexpensive way of ensuring that all of the analyzed beads carry DNA, without affecting the sequence quality [12]. Here, we present a method for selection and enrichment of beads with correct products on the surface, thus removing beads carrying unspecific sequences, which were not removed by standard gel purification protocols. This method greatly increases the fraction of informative reads obtained, thus improving the amount of high quality data generated in each sequencing run nearly three-fold.

## Results

### Experimental overview

**Figure 1:**
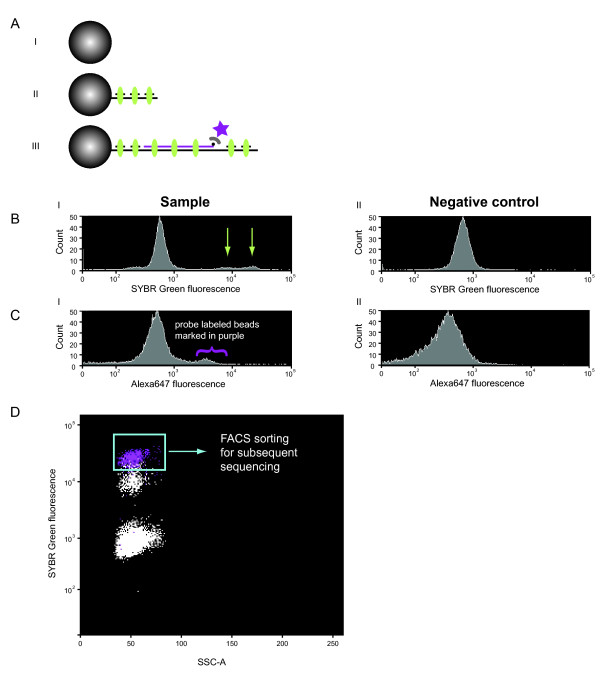
**Dual labelling strategy for FACS analysis**. (A) Schematic diagram of an emPCR sample labelled with random hexamers and SYBR Green in combination with a biotin-conjugated target-specific probe and streptavidin-conjugated Alexa647. The sample contains (I) non-fluorescent naked beads, (II) unspecific product-carrying beads displaying SYBR Green labelling (green ellipse) and (III) beads carrying amplicon target product displaying strong SYBR Green labelling and Alexa647 (purple star) labelling. (B) FACS analysis revealed two populations of DNA-covered SYBR Green fluorescent beads (arrows) in the sample (I) and a SYBR Green non-fluorescent population in both the sample and the negative control sample containing labelled naked beads (II). (C) An Alexa647 fluorescent bead population (purple brackets) with an amplified gene fragment on their surface is evident in the sample (I), while the negative control only showed the Alexa647 negative population that was also present in the sample (II). (D) FACS dotplot of the labelled sample demonstrates the distribution of SYBR Green signals, with an overlap at the higher intensities with beads that also carried the Alexa647 target-specific probe (purple dots). Beads in the square brackets were collected for sequencing by the Roche/454.

In efforts to perform ultra-deep amplicon sequencing of the hypervariable Dog Leukocyte Antigen (DLA) target using the Roche/454 Genome Sequencer we have developed a strategy based on dual-labelling of emulsion PCR (emPCR) beads and fluorescence activated cell sorting (FACS) to isolate beads carrying the target sequence on their surface as described below and illustrated in Figure 1.

The aim was to eliminate the large amounts of unspecific products present in many amplicon libraries, which are difficult to fully remove by gel or SPRI-based (eg AMPure) purification and take up precious sequencing capacity. We have achieved this by using SYBR Green to label all types of DNA-carrying beads and a target-specific probe facilitating Alexa647 detection and collection of beads carrying the target sequence.

### FACS sample preparation

**Figure 2:**
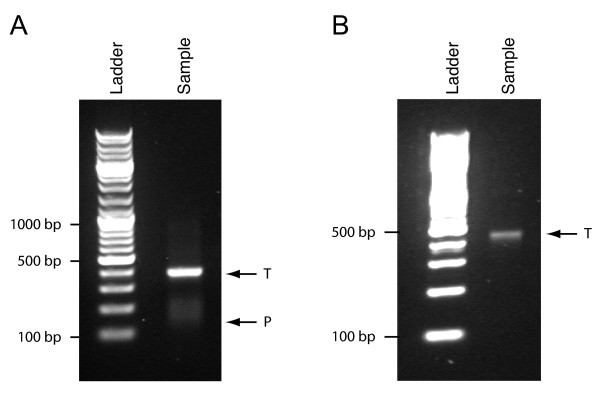
**PCR-amplified library before and after gel purification**. (A) Gel image of library prior to gel purification shows that in addition to the target fragments of ~400 bp, large frequencies of 100-200 bp unspecific products were present. (B) Gel image of library after gel purification shows no visible short unspecific products left in the library.

The analyzed sample consisted of an amplicon library prepared from 34 individuals. Amplification of the library resulted in significant formation of unspecific products, as shown in Figure 2A, so the library was purified by size separation with gel electrophoresis which, according to the manufacturer, offers best control of fragment size selection hence giving rise to the best sequencing result. The purified material, which appeared to be free from short unspecific products according to gel analysis (Figure 2B) was used for emulsion PCR and split into two batches for the comparative analysis. The control sample was processed according to the standardized Genome Sequencer FLX procedure, involving enrichment of the DNA-covered beads using Streptavidin-covered magnetic beads to capture the biotinylated protruding strands. The parallel emPCR sample was labelled as outlined above.

Flow cytometric analysis of the labelled sample showed a major peak representing naked beads that had failed to capture and amplify a DNA sequence while the DNA-covered, SYBR Green positive beads formed two minor populations with strong and weak positive SYBR Green fluorescence, respectively, as indicated by green arrows in Figure 1B. Beads with strong Alexa647 fluorescence, which thus carried an amplified target gene fragment on their surface, are indicated by purple brackets in Figure 1C. These beads are also depicted as purple dots in Figure 1D, which illustrates the SYBR Green fluorescence and SSC-A (Side Scatter Area) of the beads. Clearly, beads that yield positive Alexa647 signals also give rise to strong SYBR Green signals. In contrast, beads displaying weak positive SYBR Green signals show no Alexa647 fluorescence. Thus, the level of SYBR Green fluorescence is correlated to the amount of DNA on the bead surface and was used for guidance when setting the FACS sort gate on the Alexa647 parameter. The beads that yielded Alexa647 fluorescence signals (5.5% of the beads in total), and thus carried an amplified target gene fragment on their surface were sorted out for subsequent sequencing (see the square gate in Figure 1D). An additional experiment using FACS probe labelling was conducted confirming the previous results (see below).

### Sequence analysis

**Figure 3:**
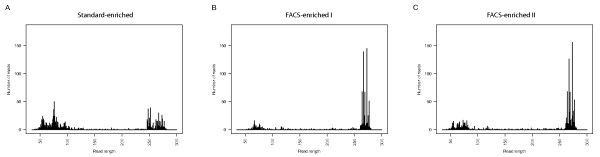
**Sequence read length distribution of a standard-enriched sample and two FACS-enriched samples**. Read length distribution plots of (A) a control sample prepared according to the standard Roche/454 procedure and (B) FACS-enriched sample I, (C) FACS-enriched duplicate sample II labelled only with gene-specific probe. All preparations also included gel purification.

The average sequence length was greatly improved by FACS enrichment (as shown by the read-length distributions from the Genome Sequencer runs for the control and FACS-sorted samples in Figure 3). Interestingly, despite thorough purification by gel electrophoresis, short unspecific fragments still accounted for a large proportion of the standard-enriched control sample library (Figure 3A). Just 41% of the reads in the control sample were longer than 200 bp, and of these 63% (25% of the total) contained the target gene sequence. In contrast, in the FACS-enriched sample many of the short fragments had been removed (Figure 3B); 69% of the reads were longer than 200 bp and 97% of these (67% of the total) contained the target gene sequence. The additional FACS sorted sample demonstrated that 75% out of all reads exceeded 200 bp and 72% out of all reads contained the target sequence. Thus, overall our FACS sorting protocol gave a nearly three-fold increase in the fraction of target sequence reads obtained.

**Figure 4:**
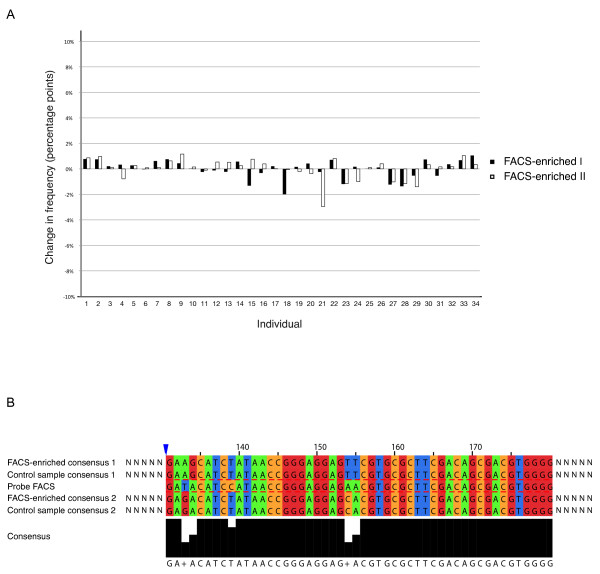
**Sequence bias analysis**. (A) Differences in sequence read frequencies between the control sample and the two FACS-enriched samples, for each individual sequenced. (B) Example of consensus sequences from the FACS probe region obtained from a FACS-enriched sample and the control sample of individual 27. The same genotypes were present in both samples.

To assess if any sequence bias was introduced by the FACS enrichment approach we calculated the change in frequencies of sequence counts for each individual (as indicated by their respective tags) in the two FACS-enriched samples as compared to the standard-enriched control sample (Figure 4A). Only minor differences were observed; the largest variations in frequency was for individual 17 and 21 where the frequencies were 2 and 3 percentage points higher in the FACS-sorted sample than in the control sample, respectively. However, for the corresponding individual in the other FACS sorted sample this change was merely 0,1 and 0,2 percentage points. For all other individuals the corresponding changes were approximately 1 percentage point or lower. Hence, the slight differences in frequency observed appear to be of stochastic nature.

We further analyzed the genotypes and found that all genotypes were represented in both FACS-enriched and control samples. Up to five mismatches between FACS probe and target sequences was observed. An example is shown in Figure 4B, where the two consensus sequences from the FACS-sorted and control samples for individual 27 are depicted. The same genotypes, with three or five mismatches against the target-specific probe, were detected in both the FACS-enriched and control sample.

To investigate whether similarity to FACS probe sequence had an effect on the enrichment efficiency of particular genotypes, the frequency of each probe region sequence present for every individual in the FACS sorted sample and the control sample, was calculated (see Additional file 1). The average differences in genotype frequencies between the FACS-enriched sample and the control sample was 10%. Many samples had changes below 5% and investigation of individuals with changes above 5% showed that there was no trend of genotypes with a higher sequence identity to the probe having a higher frequency in the FACS-enriched sample, than in the control sample. Thus, the FACS enrichment protocol does not affect what amplicon sequences are present in the amplicon library after FACS sorting.

## Discussion

We have previously demonstrated that a generic fluorescently labelled FACS probe common to all 454 sequences (i.e. A/B linker) can be effectively used to identify DNA-carrying beads to simplify the titration step in Roche/454 sequencing and (more importantly) facilitate enrichment of pure samples with DNA-carrying beads produced by a shotgun sample preparation procedure [12]. In our current work we have taken this method further by showing that beads carrying a specific target DNA sequence can be collected for sequencing using flow cytometry and hence beads carrying an unspecific product that includes A or B linkers can be removed. This could be used with unparalleled efficiency to enhance sequence output of problematic amplicon libraries.

In the technique presented here we exploit an additional feature of FACS (its ability to detect more than one fluorescent label) to increase the resolution of the procedure and remove the high numbers of non-target amplicons. This approach could be used for most cases of amplicon deep sequencing, such as analysis of 16S rRNA, mtDNA, cancer genes etc. The probe-mediated FACS enrichment protocol, using a probe tolerating at least five mismatches, provides sufficient flexibility to enrich beads carrying DLA target sequence (with significant between-amplicon variation). The results also show that the relative frequencies of sequences from different individuals in the amplicon library were not affected by FACS enrichment. Further we show that genotypes with a probe region sequence very similar to the FACS probe were not favoured by the FACS enrichment protocol over genotypes with up to five mismatches against the FACS probe.

The beads carrying the target sequence could be detected using solely the target-specific probe. However, the dual labelling system confirms the efficiency of the target-specific labelling. Further, some stickiness of the probe to naked and unspecific product- carrying beads was observed (data not shown), probably due to the length of the target-specific probe, and use of two labels greatly increases the resolution and scope for optimising the FACS sort gate positions.

Further, this report illustrates the possibility of fishing out specific sequences carried on beads using FACS, a feature that could be used for colour-coded MID (Multiplex Identifier, Roche/454 tag)-specific FACS enrichment, in order to load and sequence optimal numbers of beads carrying each MID-tag. The value of this would be the ability to compensate for differences in amplification efficiency between members of a MID library.

Moreover, the results show that FACS-based quantification of DNA contents, and thus active selection of beads carrying long DNA fragments, could be used to increase average sequence read lengths in Roche/454 sequencing runs.

Another future possibility would be to enhance sort speeds to further increase throughput. In order to maximise yield we used a modest sorting speed, of 1000 beads/s, but with a maximal sorting speed of 70,000 beads/s FACS enrichment of beads for large-scale sequencing would not be a rate-limiting step in the sample preparation procedure for Roche/454 Sequencing.

## Conclusions

Our results demonstrate that the number of informative sequence reads can be substantially increased by using gene-specific FACS sorting to remove most beads carrying amplified unspecific fragments on their surface, prior to sequencing using a Roche/454 Genome Sequencer.

Using a biotinylated 50 mer FACS probe and streptavidin-conjugated Alexa647 florophore, beads with an amplified target gene product on their surface could be collected by FACS sorting for subsequent sequencing using the Roche/454 Genome Sequencer. This procedure gives a nearly three-fold increase in the fraction of reads with the target amplicon sequence. The method also allows for several mismatches between the sequence and the FACS probe and does not significantly affect the distribution of sequence reads over different individuals or genotypes obtained.

## Methods

### Sample preparation

An amplicon library of 34 individuals was prepared by PCR of the Dog Leukocyte Antigen gene DLA-DRB1 exon 2 (target size: 303 bp) [13], introducing identifying tags and the common Roche/454 A/B FLX sequences. The individuals had previously been shown to be heterozygous for this gene by traditional Sanger sequencing. The library was analyzed by agarose gel electrophoresis and ethidium bromide staining. Approximately 400-bp long fragments were extracted by gel purification and the product was then analysed by agarose gel electrophoresis and GelRed (Biotium, Inc., Hayward, CA, USA) staining. EmPCR and strand-separation were carried out according to instructions of the manufacturer of the sequencing system (Roche). Briefly, an emulsion was formed by shaking "mock buffer" with mineral oil, then PCR reagents, the DNA library and beads were shaken into the emulsion. Following clonal amplification of library fragments on beads, the emulsion was broken and beads were collected by centrifugation. For labelling and subsequent FACS enrichment, the protruding DNA strands were removed by washing the collected beads in 0.1 M NaOH according to the manufacturer's protocol. For comparison, a control emPCR reaction mixture was prepared from the same library, and unspecific enrichment of DNA-covered beads was carried out, using streptavidin-covered beads, again according to the manufacturer's instructions.

### emPCR product labelling

For detecting beads carrying the amplified gene fragment by FACS, a biotinylated 50 mer was designed to match the central part of the amplicon product and still allow for some mismatches in the hypervariable DLA target sequence. To investigate whether the amount of DNA on the beads could be visualised by FACS, beads were also labelled with random hexamers and SYBR Green.

Dual labelling was carried out by adding 20 pmol random hexamers and 20 pmol biotinylated gene-specific 50 mer probe, both from Eurofins MWG Operon (Ebersberg, Germany) in a total volume of 100 μl 1× Annealing Buffer (1×AB, Roche, Basel, Switzerland) and applying the following conditions: 1 min at 95°C, then decreasing to 25°C over 10 min. 1 μl 50 × SYBR Green I Nucleic Acid Gel Stain (SYBR Green, Molecular Probes Inc., Eugene, OR, USA) and 4 μl streptavidin-conjugated Alexa647 dye (0.2 mg/ml, Invitrogen, Carlsbad, CA, USA) were then added to each sample, and the resulting mixture was incubated for 10 min at room temperature. Samples were then transferred to 5 ml Polystyrene Round-Bottom FACS Tubes (BD Biosciences, Franklin Lakes, NJ, USA) and diluted by adding 500 μl phosphate-buffered saline (PBS) containing 0.1% Pluronic F108 NF surfactant (PBSP) (BASF Corporation, Mount Olive, NJ, USA). As negative FACS control, unreacted beads were labelled in the same manner.

### FACS enrichment

FACS enrichment of beads carrying an amplified gene fragment on their surface was achieved by sorting out beads that were Alexa647 positive using a FACSAria instrument (Beckton Dickinson, San Jose, California). Beads were transferred to Ultrafree^®^-MC Centrifugal Filter Units (Millipore, Billerica, MA, 0.65 mm pore size) and washed twice in 140 μl 0.1 M NaOH for 40 s and twice in 250 μl 95°C 1×AB for 40 s, followed by re-suspension in 1×AB.

### Sequencing

FACS-enriched and standard-enriched control samples were sequenced in separate 1/16-lanes of a picotiter plate according to the instructions of the sequencing system.

### Sequence analysis

Sequences were sorted by individual using an in-house developed Perl script, aligned using Muscle [14], Jalview version 2 [15] was employed for visualisation and analysis of the alignments.

## Competing interests

The authors declare that they have no competing interests.

## Authors' contributions

JS participated in the design of the study, collection of data, data analysis and interpretation and manuscript writing; MN participated in the design of the study, collection of data, data analysis and interpretation; AA participated in the design of the study, data analysis and interpretation; and JL participated in the design of the study and writing of the manuscript. All authors read and approved the final manuscript.

## Supplementary Material

Additional file 1 **Genotype frequency in standard-enriched sample and FACS-enriched sample**. The data provided represent the frequency of genotypes of the different individuals in the standard-enriched sample and the FACS-enriched sample. Frequencies of individuals with less than ten sequence reads (N/A) and individuals with two different genotypes but with identical consensus sequences in the probe region were omitted. Click here for file
