# Supplementary material for: Gene-specific FACS sorting method for target selection in high-throughput amplicon sequencing
Source: BMC Genomics. 2010 Feb 26;11:140. doi: 10.1186/1471-2164-11-140 (PMC2842249; doi:10.1186/1471-2164-11-140)
Supplement: Additional file 1 — Genotype frequency in standard-enriched sample and FACS-enriched sample. The data provided represent the frequency of genotypes of the different individuals in the standard-enriched sample and the FACS-enriched sample. Frequencies of individuals with less than ten sequence reads (N/A) and individuals with two different genotypes but with identical consensus sequences in the probe region were omitted. [file 1471-2164-11-140-S1.PDF]

Individual	Probe region sequence	# Mismatches	Frequency in standard-enriched sample	Frequency in FACS-enriched sample	Change
1	A	0	0,49	0,62	-0,13
	B	3	0,51	0,38	
2	A	3	-	-	N/A
	-	-	-	-	
3	A	4	0,44	0,28	0,17
	B	3	0,56	0,72	
4	A	0	-	-	N/A
	-	-	-	-	
5	N/A	N/A	N/A	N/A	N/A
6	N/A	N/A	N/A	N/A	N/A
7	A	2	0,29	0,33	-0,05
	B	3	0,71	0,67	
8	A	0	0,58	0,52	0,07
	B	5	0,42	0,48	
9	N/A	N/A	N/A	N/A	N/A
10	N/A	N/A	N/A	N/A	N/A
11	A	3	0,60	0,60	0,00
	B	2	0,40	0,40	
12	N/A	N/A	N/A	N/A	N/A
13	N/A	N/A	N/A	N/A	N/A
14	N/A	N/A	N/A	N/A	N/A
15	A	3	0,33	0,18	0,15
	B	2	0,67	0,82	
16	A	3	-	-	N/A
	-	-	-	-	
17	A	0	0,69	0,50	0,19
	B	4	0,31	0,50	
18	A	3	0,58	0,58	0,00
	B	3	0,42	0,42	
19	A	3	0,50	0,58	-0,08
	B	2	0,50	0,42	
20	A	2	-	-	N/A
	-	-	-	-	
21	A	0	0,53	0,41	0,12
	B	2	0,47	0,53	
22	A	0	0,51	0,36	0,15
	B	2	0,49	0,64	
23	A	0	0,72	0,82	-0,10
	B	4	0,28	0,18	
24	A	3	0,53	0,50	0,03
	B	3	0,47	0,50	
25	A	3	0,50	0,60	-0,10
	B	2	0,50	0,40	
26	N/A	N/A	N/A	N/A	N/A
27	A	5	0,46	0,81	-0,35
	B	3	0,54	0,19	
28	A	3	0,82	0,74	0,08
	B	3	0,18	0,26	
29	A	0	0,59	0,50	0,09
	B	4	0,41	0,50	
30	A	3	0,45	0,45	-0,01
	B	2	0,55	0,55	
31	A	3	0,38	0,24	0,15
	B	2	0,62	0,76	
32	A	3	0,71	0,50	0,21
	B	1	0,29	0,50	
33	A	3	0,45	0,52	-0,07
	B	2	0,55	0,48	
34	A	0	0,66	0,67	0,00
	B	3	0,34	0,33	
